# The genome sequence of the turban top shell, *Gibbula magus *(Linnaeus, 1758)

**DOI:** 10.12688/wellcomeopenres.18792.1

**Published:** 2023-01-23

**Authors:** Patrick Adkins, Joanna Harley, Teresa Darbyshire, Anna Holmes, Marine Biological Association Genome Acquisition Lab, Darwin Tree of Life Barcoding collective, Wellcome Sanger Institute Tree of Life programme, Wellcome Sanger Institute Scientific Operations: DNA Pipelines collective, Tree of Life Core Informatics collective, Kesella Scott-Somme, Nova Mieskowska, Darwin Tree of Life Consortium

**Affiliations:** 1 Marine Biological Association, Plymouth, UK; 2 Amgueddfa Cymru, Cardiff, Wales, UK

**Keywords:** Gibbula magus, turban top shell, genome sequence, chromosomal, Trochida

## Abstract

We present a genome assembly from an individual *Gibbula magus* (the turban top shell; Mollusca; Gastropoda; Trochida; Trochidae). The genome sequence is 1,470 megabases in span. Most of the assembly is scaffolded into 18 chromosomal pseudomolecules. The mitochondrial genome has also been assembled and is 16.1 kilobases in length. Gene annotation of this assembly on Ensembl identified 41,167 protein coding genes.

## Species taxonomy

Eukaryota; Metazoa; Spiralia; Lophotrochozoa; Mollusca; Gastropoda; Vetigastropoda; Trochida; Trochoidea; Trochidae; Cantharidinae; *Gibbula*; *Gibbula magus* (Linnaeus, 1758) (NCBI:txid703304).

## Background

*Gibbula magus* is a marine gastropod mollusc in the Trochidae family, known as topshells ( NBN Atlas, 2021). They are usually sublittoral, and found on muddy sandy or gravel, on algae or under stones, where they feed on microphytes ( Smith, 2015). They can be found in the intertidal zone at extreme low spring tides and down to depths of 70 m ( Wilson, 2017). In Great Britain, *G. magus* is seldom found on the east coast, occurring almost exclusively on the south and west coasts, and on all coasts in Ireland ( de Kluijver *et al.*, 2000; NBN Atlas, 2021). The breeding times of *G. magus* varies geographically; June at Roscoff and spring and autumn at Plymouth. Fertilisation happens externally and there is a brief free-living trochophore larval stage, however, little else is known about these early life stages ( Smith, 2015). The fringe present on the body of *G. magus* is thought to protect against detritus and may be able to sense poor water conditions ( Smith, 2015).

The *Gibbula* genus can be difficult to identify due to high variability in the shell morphology (the main identifying feature) unspecific or missing type material and vague original descriptions ( Affenzeller *et al*., 2017). Adult *G. magus* are the largest of the trochid species found in UK waters. They have a flattened spire and a large umbilicus, and diagonal lines of pink coloured dots along the whorls of the shell. The taxonomic divides of the Trochidae family have also been investigated, with changes to the groupings and names of several species ( Affenzeller *et al.*, 2017; Anistratenko, 2005). Quality genomic data can add more information and detail for how these decisions are made, and support the work of taxonomists ( Coates *et al.*, 2018). Here we present a chromosomally complete genome sequence for *G. magus*, based on a specimen from Gann Bay, Pembrokeshire, UK.

## Genome sequence report

**Figure 1.**
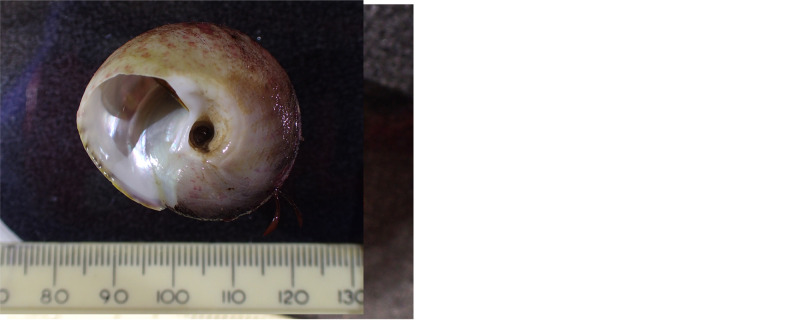
Photographs of the *Gibbula magus* (xgGibMagu1) specimen used for genome sequencing.

The genome was sequenced from a *Gibbula magus* specimen ( Figure 1) collected from Gann Bay, Pembrokeshire, UK (latitude 51.71, longitude –5.17). A total of 35-fold coverage in Pacific Biosciences single-molecule HiFi long reads and 36-fold coverage in 10X Genomics read clouds was generated. Primary assembly contigs were scaffolded with chromosome conformation Hi-C data. Manual assembly curation corrected 477 missing joins or misjoins and removed 93 haplotypic duplications, reducing the assembly length by 4.9% and the scaffold number by 54.52%, and increasing the scaffold N50 by 3.38%.

**Figure 2.**
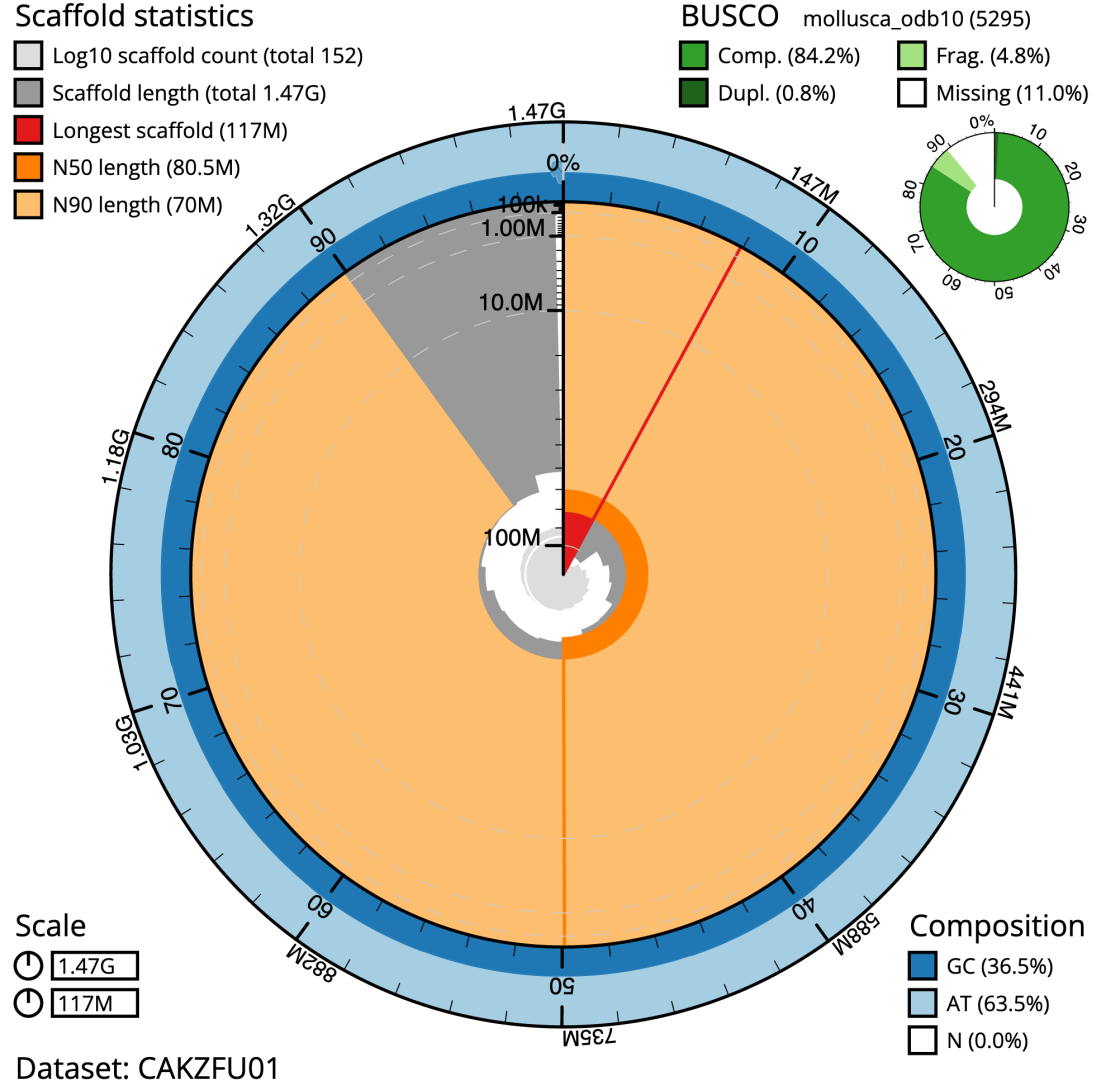
Genome assembly of *Gibbula magus*, xgGibMagu1.1: metrics. The BlobToolKit Snailplot shows N50 metrics and BUSCO gene completeness. The main plot is divided into 1,000 size-ordered bins around the circumference with each bin representing 0.1% of the 1,470,404,880 bp assembly. The distribution of scaffold lengths is shown in dark grey with the plot radius scaled to the longest scaffold present in the assembly (117,468,434 bp, shown in red). Orange and pale-orange arcs show the N50 and N90 scaffold lengths (80,454,948 and 69,970,916 bp), respectively. The pale grey spiral shows the cumulative scaffold count on a log scale with white scale lines showing successive orders of magnitude. The blue and pale-blue area around the outside of the plot shows the distribution of GC, AT and N percentages in the same bins as the inner plot. A summary of complete, fragmented, duplicated and missing BUSCO genes in the mollusca_odb10 set is shown in the top right. An interactive version of this figure is available at https://blobtoolkit.genomehubs.org/view/xgGibMagu1.1/dataset/CAKZFU01/snail.

The final assembly has a total length of 1,470.4 Mb in 151 sequence scaffolds with a scaffold N50 of 80.5 Mb ( Table 1). Most (99.51%) of the assembly sequence was assigned to 9 chromosomal-scale scaffolds. Chromosome-scale scaffolds confirmed by the Hi-C data are named in order of size ( Figure 2– Figure 5; Table 2). While not fully phased, the assembly deposited is of one haplotype. Contigs corresponding to the second haplotype have also been deposited. The mitochondrial genome was also assembled.

**Table 1.** Genome data for *Gibbula magus*, xgGibMagu1.1.

Project accession data		
Assembly identifier	xgGibMagu1.1	
Species	*Gibbula magus*	
Specimen	xgGibMagu1	
NCBI taxonomy ID	703304	
BioProject	PRJEB51161	
BioSample ID	SAMEA8717440	
Isolate information		
Assembly metrics *		*Benchmark*
Consensus quality (QV)	52.2	*≥ 50*
*k*-mer completeness	99.98	*≥ 95%*
BUSCO **	C:84.2%[S:83.4%,D:0.8%],F:4.8%,M:11.0%,n:5,295	*C ≥ 95%*
Percentage of assembly mapped to chromosomes	99.51%	*≥ 95%*
Organelles	mitochondrial genome assembled	*complete* * single alleles*
Raw data accessions		
PacificBiosciences SEQUEL II	ERR9127937–ERR9127939	
10X Genomics Illumina	ERR8974924–ERR8974927	
Hi-C Illumina	ERR8974928	
PolyA RNA-Seq Illumina	ERR10123686	
Genome assembly		
Assembly accession	GCA_936450465.1	
*Accession of alternate haplotype*	GCA_936439675.1	
Span (Mb)	1,470.4	
Number of contigs	1,001	
Contig N50 length (Mb)	3.4	
Number of scaffolds	151	
Scaffold N50 length (Mb)	80.5	
Longest scaffold (Mb)	117.5	

* Assembly metric benchmarks are adapted from column VGP-2020 of “Table 1: Proposed standards and metrics for defining genome assembly quality” from ( Rhie *et al.*, 2021). ** BUSCO scores based on the mollusca_odb10 BUSCO set using v5.3.2. C = complete [S = single copy, D = duplicated], F = fragmented, M = missing, n = number of orthologues in comparison. A full set of BUSCO scores is available at https://blobtoolkit.genomehubs.org/view/xgGibMagu1.1/dataset/CAKZFU01/busco.

**Figure 3.**
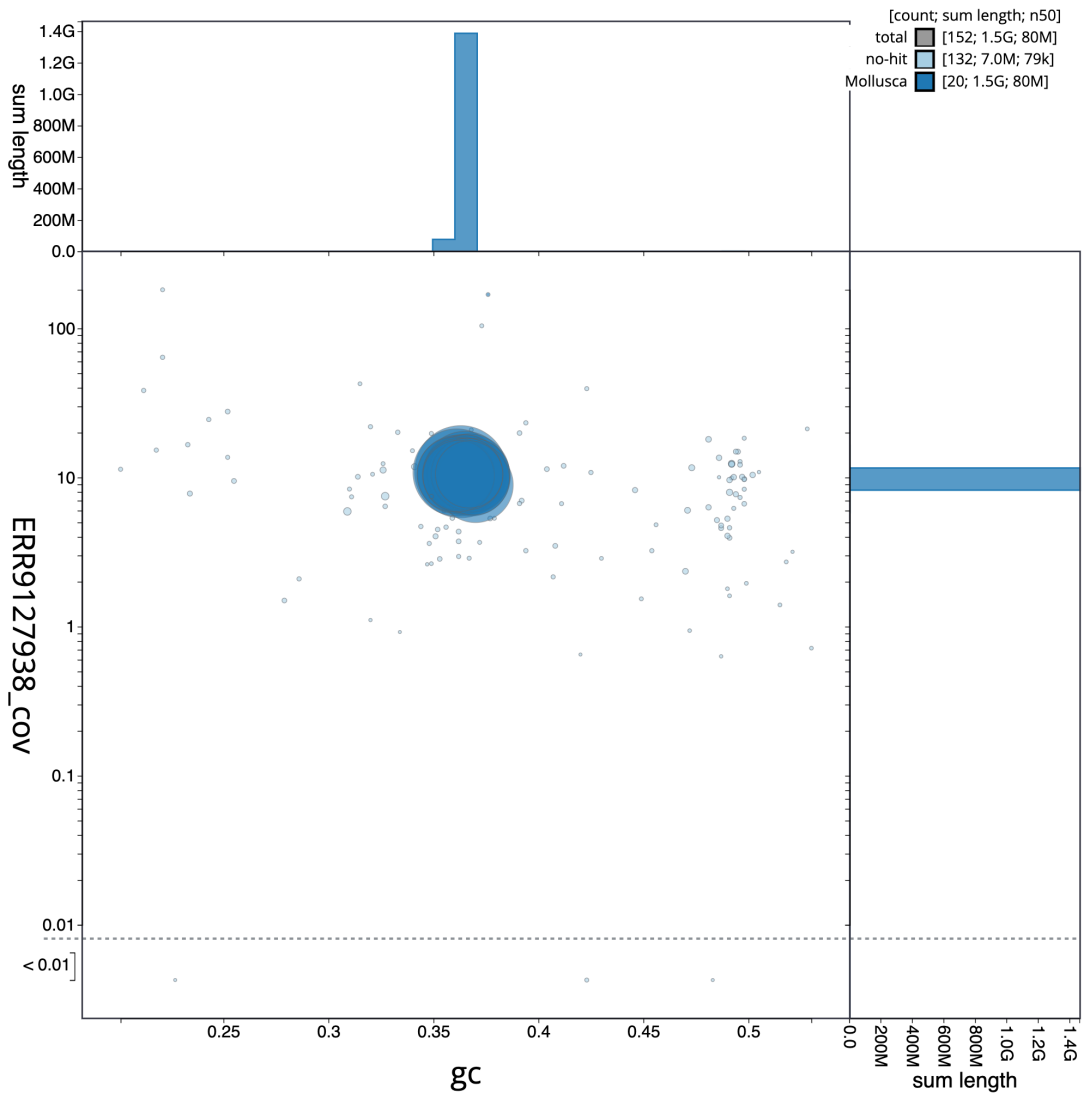
Genome assembly of *Gibbula magus*, xgGibMagu1.1: GC coverage. BlobToolKit GC-coverage plot. Sequences are coloured by phylum. Circles are sized in proportion to scaffold length. Histograms show the distribution of scaffold length sum along each axis. An interactive version of this figure is available at https://blobtoolkit.genomehubs.org/view/xgGibMagu1.1/dataset/CAKZFU01/blob.

**Figure 4.**
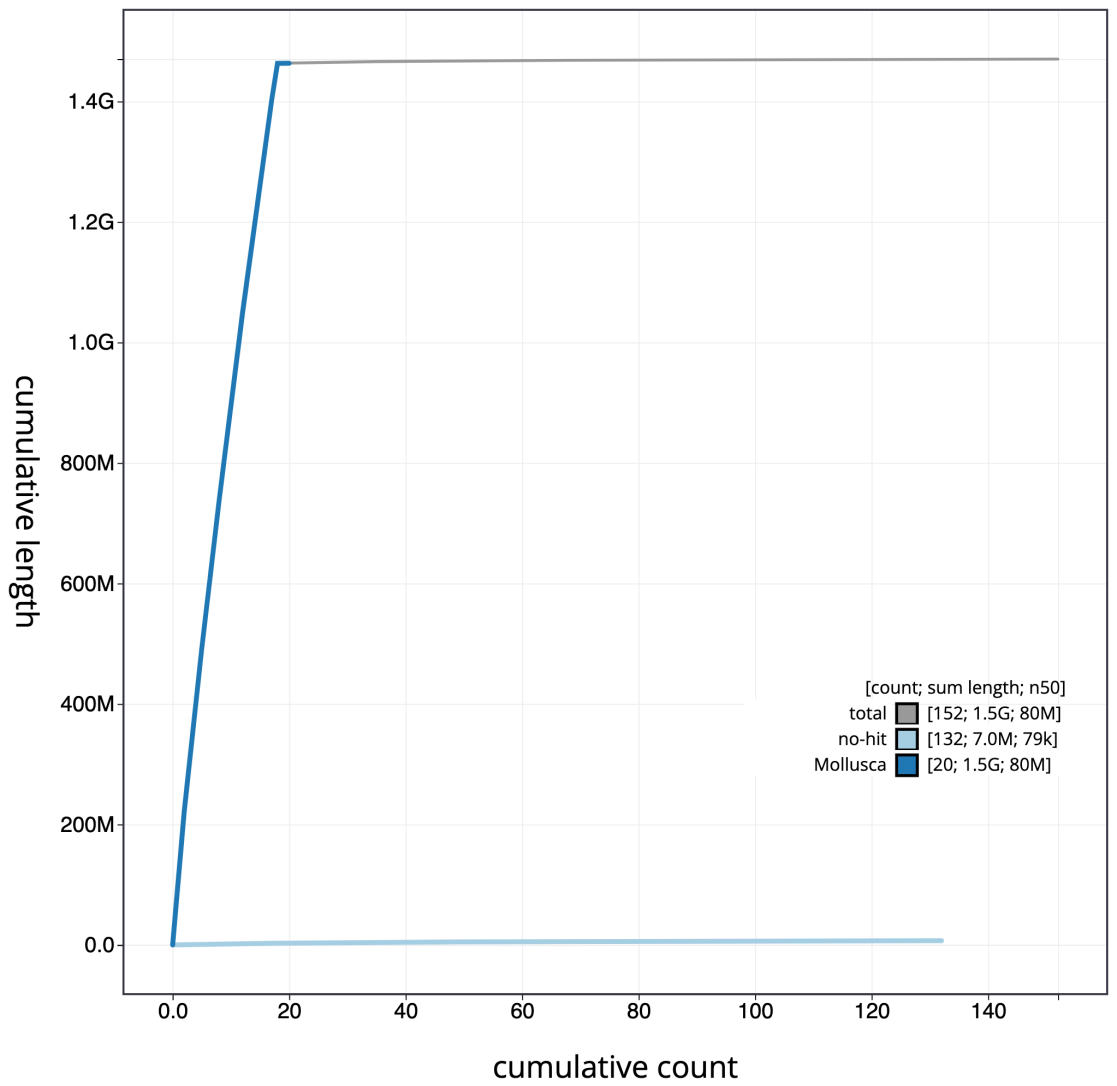
Genome assembly of *Gibbula magus*, xgGibMagu1.1: cumulative sequence. BlobToolKit cumulative sequence plot. The grey line shows cumulative length for all scaffolds. Coloured lines show cumulative lengths of scaffolds assigned to each phylum using the buscogenes taxrule. An interactive version of this figure is available at https://blobtoolkit.genomehubs.org/view/xgGibMagu1.1/dataset/CAKZFU01/cumulative.

**Figure 5.**
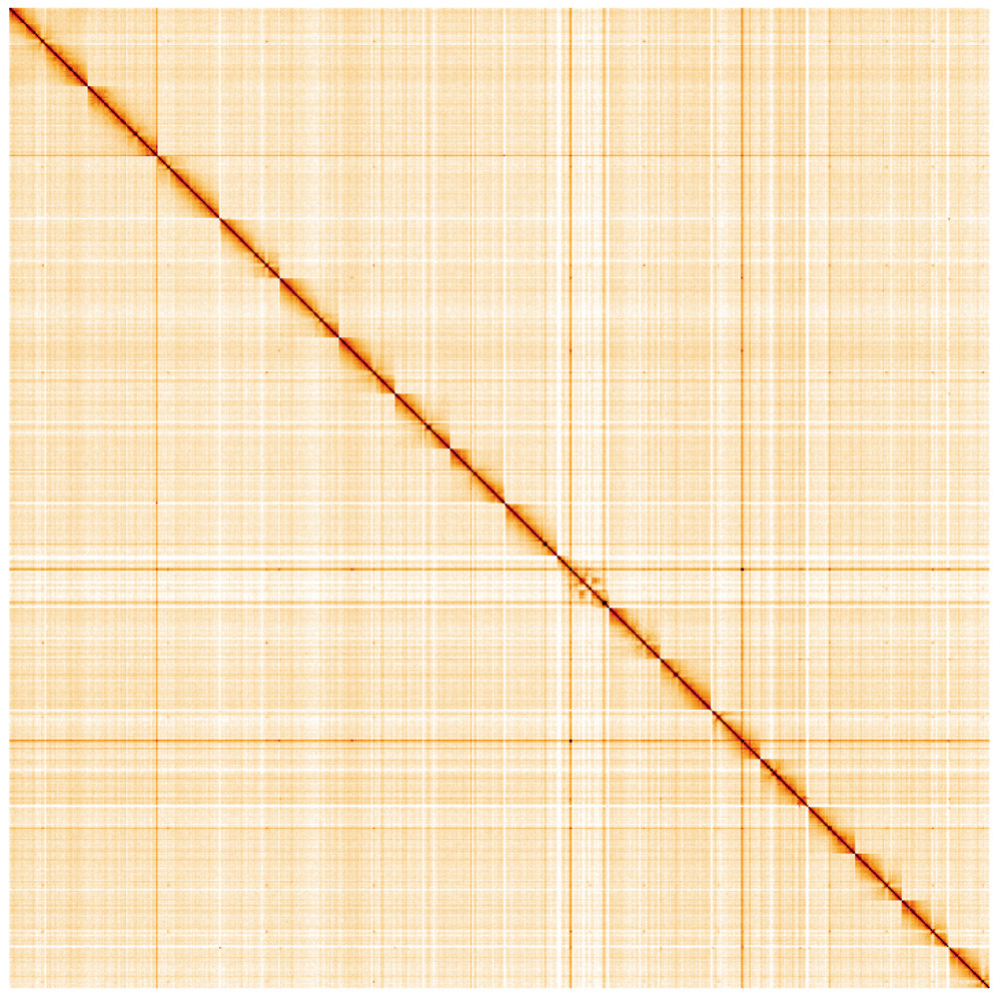
Genome assembly of *Gibbula magus*, xgGibMagu1.1: Hi-C contact map. Hi-C contact map of the xgGibMagu1.1 assembly, visualised using HiGlass. Chromosomes are given in order of size from left to right and top to bottom. An interactive version of this plot is at: https://genome-note-higlass.tol.sanger.ac.uk/l/?d=N9lkINhLTXmGpbn8oj1olQ.

**Table 2.** Chromosomal pseudomolecules in the genome assembly of *Gibbula magus*, xgGibMagu1.

INSDC accession	Chromosome	Size (Mb)	GC%
OW388289.1	1	117.47	36.3
OW388290.1	2	104.47	36.1
OW388291.1	3	92.77	36.5
OW388292.1	4	89.61	36.7
OW388293.1	5	88	36.4
OW388294.1	6	83.61	36.1
OW388295.1	7	83.18	36.8
OW388296.1	8	80.45	36.3
OW388297.1	9	78.28	36.5
OW388298.1	10	77.47	37
OW388299.1	11	77.31	36.5
OW388300.1	12	76.35	36
OW388301.1	13	73.01	36.9
OW388302.1	14	70.77	36.9
OW388303.1	15	70.18	36.3
OW388304.1	16	69.97	36.6
OW388305.1	17	69.2	36.2
OW388306.1	18	61.23	36.7
OW388307.1	MT	0.02	37.9

The assembly has a BUSCO v5.3.2 ( Manni *et al.*, 2021) completeness of 84.2% (single 83.4%, duplicated 0.8%) using the OrthoDB-v10 mollusca reference set. BUSCO loci identified as fragmented accounted for a further 4.8% of loci tested. This low BUSCO score may be due to low conservation of orthologues between *G. magus* and the molluscan species in the reference set, or underperformance of the BUSCO gene finder given the particular gene structures in this species. The assembly is validated by the other assembly quality metrics ( *k*-mer completeness 99.98%, consensus quality (QV) 52.2) shown in Table 1.

## Genome annotation report

The *G*. *magus* (GCA_936450465.1) genome assembly was annotated using BRAKER2 in the Ensembl rapid annotation pipeline ( Table 1; https://rapid.ensembl.org/Gibbula_magus_GCA_936450465.1/Info/Index). The resulting annotation includes 41,235 transcribed mRNAs from 41,167 protein-coding genes.

## Methods

### Sample acquisition and nucleic acid extraction

The collectors of the *G. magus* specimen (xgGibMagu1) used for genome sequencing were Patrick Adkins and Joanna Harley (Marine Biological Association) and Teresa Darbyshire and Anna Holmes (Amgueddfa Cymru), and the specimen was then identified by Patrick Adkins and Joanna Harley. The specimen was collected in Gann Bay, Pembrokeshire, UK (latitude 51.71, longitude –5.17). The sample was taken from sediment by hand and placed into a container, using and then snap-frozen in liquid nitrogen.

DNA was extracted at the Tree of Life laboratory, Wellcome Sanger Institute (WSI). The xgGibMagu1 sample was weighed and dissected on dry ice with tissue set aside for Hi-C sequencing. Muscle tissue was cryogenically disrupted to a fine powder using a Covaris cryoPREP Automated Dry Pulveriser, receiving multiple impacts. High molecular weight (HMW) DNA was extracted using the Qiagen MagAttract HMW DNA extraction kit. Low molecular weight DNA was removed from a 200 ng aliquot of extracted DNA using 0.8X AMpure XP purification kit prior to 10X Chromium sequencing; a minimum of 50 ng DNA was submitted for 10X sequencing. HMW DNA was sheared into an average fragment size between 12–20 kb in a Megaruptor 3 system with speed setting 30. Sheared DNA was purified by solid-phase reversible immobilisation using AMPure PB beads with a 1.8X ratio of beads to sample to remove the shorter fragments and concentrate the DNA sample. The concentration of the sheared and purified DNA was assessed using a Nanodrop spectrophotometer and Qubit Fluorometer and Qubit dsDNA High Sensitivity Assay kit. Fragment size distribution was evaluated by running the sample on the FemtoPulse system.

RNA was extracted from muscle tissue of xgGibMagu1 in the Tree of Life Laboratory at the WSI using TRIzol, according to the manufacturer’s instructions. RNA was then eluted in 50 μl RNAse-free water and its concentration RNA assessed using a Nanodrop spectrophotometer and Qubit Fluorometer using the Qubit RNA Broad-Range (BR) Assay kit. Analysis of the integrity of the RNA was done using Agilent RNA 6000 Pico Kit and Eukaryotic Total RNA assay.

### Sequencing

Pacific Biosciences HiFi circular consensus and 10X Genomics read cloud DNA sequencing libraries were constructed according to the manufacturers’ instructions. Poly(A) RNA-Seq libraries were constructed using the NEB Ultra II RNA Library Prep kit. DNA and RNA sequencing was performed by the Scientific Operations core at the WSI on Pacific Biosciences SEQUEL II (HiFi), Illumina NovaSeq 6000 (RNA-Seq and 10X) instruments. Hi-C data were also generated from muscle tissue of xgGibMagu1 using the Arima v2 kit and sequenced on the Illumina NovaSeq 6000 instrument.

### Genome assembly

Assembly was carried out with Hifiasm ( Cheng *et al.*, 2021) and haplotypic duplication was identified and removed with purge_dups ( Guan *et al.*, 2020). One round of polishing was performed by aligning 10X Genomics read data to the assembly with Long Ranger ALIGN, calling variants with freebayes ( Garrison & Marth, 2012). The assembly was then scaffolded with Hi-C data ( Rao *et al.*, 2014) using YaHS ( Zhou *et al.*, 2022). The assembly was checked for contamination and corrected as described previously ( Howe *et al.*, 2021). Manual curation was performed using HiGlass ( Kerpedjiev *et al.*, 2018) and Pretext ( Harry, 2022). The mitochondrial genome was assembled using MitoHiFi ( Uliano-Silva *et al.*, 2022), which performed annotation using MitoFinder ( Allio *et al.*, 2020). The genome was analysed and BUSCO scores were generated within the BlobToolKit environment ( Challis *et al.*, 2020). Table 3 contains a list of all software tool versions used, where appropriate.

**Table 3.** Software tools used.

Software tool	Version	Source
BlobToolKit	3.3.10	Challis *et al.*, 2020
freebayes	1.3.1-17- gaa2ace8	Garrison & Marth, 2012
Hifiasm	0.15.3-r339	Cheng *et al.*, 2021
HiGlass	1.11.6	Kerpedjiev *et al.*, 2018
Long Ranger ALIGN	2.2.2	https://support.10xgenomics.com/genome-exome/ software/pipelines/latest/advanced/other-pipelines
MitoHiFi	2.0	Uliano-Silva *et al.*, 2022
PretextView	0.2.x	Harry, 2022
purge_dups	1.2.3	Guan *et al.*, 2020
YaHS	yahs-1.1.91eebc2	Zhou *et al.*, 2022

### Genome annotation

The Ensembl gene annotation system ( Aken *et al.*, 2016) was used to generate annotation for the *G. magus* assembly (GCA_936450465.1). Annotation was created primarily through alignment of transcriptomic data to the genome, with gap filling via protein to-genome alignments of a select set of proteins from UniProt ( UniProt Consortium, 2019).

### Ethics/compliance issues

The materials that have contributed to this genome note have been supplied by a Darwin Tree of Life Partner. The submission of materials by a Darwin Tree of Life Partner is subject to the Darwin Tree of Life Project Sampling Code of Practice. By agreeing with and signing up to the Sampling Code of Practice, the Darwin Tree of Life Partner agrees they will meet the legal and ethical requirements and standards set out within this document in respect of all samples acquired for, and supplied to, the Darwin Tree of Life Project. Each transfer of samples is further undertaken according to a Research Collaboration Agreement or Material Transfer Agreement entered into by the Darwin Tree of Life Partner, Genome Research Limited (operating as the Wellcome Sanger Institute), and in some circumstances other Darwin Tree of Life collaborators.

## Data Availability

European Nucleotide Archive: *Gibbula magus* (turban top shell). Accession number PRJEB51161; https://www.ebi.ac.uk/ena/browser/view/PRJEB51161 ( Wellcome Sanger Institute, 2022). The genome sequence is released openly for reuse. The *Gibbula magus* genome sequencing initiative is part of the Darwin Tree of Life (DToL) project. All raw sequence data and the assembly have been deposited in INSDC databases. Raw data and assembly accession identifiers are reported in Table 1.

## Author information

Members of the Marine Biological Association Genome Acquisition Lab are listed here: https://doi.org/10.5281/zenodo.4783604.

Members of the Darwin Tree of Life Barcoding collective are listed here: https://doi.org/10.5281/zenodo.5744972.

Members of the Wellcome Sanger Institute Tree of Life programme are listed here: https://doi.org/10.5281/zenodo.6125027.

Members of Wellcome Sanger Institute Scientific Operations: DNA Pipelines collective are listed here: https://doi.org/10.5281/zenodo.5746904.

Members of the Tree of Life Core Informatics collective are listed here: https://doi.org10.5281/zenodo.6125046.

Members of the Darwin Tree of Life Consortium are listed here: https://doi.org/10.5281/zenodo.5638618.
