# Supplementary material for: The genome sequence of the turban top shell, Gibbula magus (Linnaeus, 1758)
Source: Wellcome Open Res. 2023 Jan 23;8:35. [Version 1] doi: 10.12688/wellcomeopenres.18792.1 (PMC10394393; doi:10.12688/wellcomeopenres.18792.1)
Supplement: Peer review history

# Peer review: Reviewer response for version 1

**DOI:** 10.21956/wellcomeopenres.20837.r63422

**Published:** 2023-08-01

**Authors:** Qinggang Xue

**Affiliations:** 1 Zhejiang Wanli University, Ningbo, Zhejiang, China

The authors reported a high quality chromosome –level genome assembly for the *Gibbula magus*, it would be useful for supporting the work of taxonomists and evolution analysis .The manuscript is acceptable after minor revision.

 Minor suggestions

1. In the abstract, the mitochondrial genome were mentioned, however, the sequence was not uploaded, at least not appeared in the data availability section.
2. In the method section, the genome prediction and annotation methods should be much clearly present.
3. Figure 1, the unit of the ruler are not marked out.

Are sufficient details of methods and materials provided to allow replication by others?

Partly

Is the rationale for creating the dataset(s) clearly described?

Yes

Are the datasets clearly presented in a useable and accessible format?

Yes

Are the protocols appropriate and is the work technically sound?

Yes

Reviewer Expertise:

Gene function, genome

I confirm that I have read this submission and believe that I have an appropriate level of expertise to confirm that it is of an acceptable scientific standard.

# Peer review: Reviewer response for version 1

**DOI:** 10.21956/wellcomeopenres.20837.r59302

**Published:** 2023-06-19

**Authors:** Iker Irisarri

**Affiliations:** 1 Centre for Molecular Biodiversity Research, Leibniz Institute for the Analysis of Biodiversity Change (LIB), Hamburg, Germany

The rationale for this new genome is clearly explained. The genome assembly and annotation pipelines represent the current gold standards and are sufficiently explained. The resulting genome is thus of high quality and it is made fully accessible to the community.

 I have only two very minor comments on the text that I feel could increase clarity:

- Abstract. The sentence “Most of the assembly is scaffolded into 18 chromosomal pseudomolecules” could be understood as the assembly consisting of 18 pseudochromosomes when it is actually a phased assembly of 9 pseudochromosomes.
- Blobtoolkit and Figure 3 are not mentioned in the text. Also, the caption of Figure 3 (“GC coverage”) could be clarified.

Are sufficient details of methods and materials provided to allow replication by others?

Yes

Is the rationale for creating the dataset(s) clearly described?

Yes

Are the datasets clearly presented in a useable and accessible format?

Yes

Are the protocols appropriate and is the work technically sound?

Yes

Reviewer Expertise:

Phylogenomics and comparative genomics

I confirm that I have read this submission and believe that I have an appropriate level of expertise to confirm that it is of an acceptable scientific standard.
